# Complex Messages in Long-Term Monitoring of Regal Fritillary (*Speyeria idalia*) (Lepidoptera: Nymphalidae) in the State of Wisconsin, USA, 1988–2015

**DOI:** 10.3390/insects8010006

**Published:** 2017-01-10

**Authors:** Ann B. Swengel, Scott R. Swengel

**Affiliations:** 909 Birch Street, Baraboo, WI 53913, USA; aswengel@jvlnet.com

**Keywords:** population monitoring, prairie, habitat management, specialist butterfly, climate, flight period, phenology, 4th of July Butterfly Count Program, butterfly survey methods, spatial synchrony

## Abstract

The regal fritillary (“regal”) (*Speyeria idalia*) is endangered in Wisconsin, USA, and declining and at risk range-wide. During 1988–2015, we surveyed 24 known regal sites and >100 areas of potential habitat in Wisconsin. We recorded 9037 individuals in 742.7 km on the peak survey per year at occupied sites. At six sites surveyed over 5–25 years, we found regal fritillaries in only one year, mostly in the latter half of the study. The three populations in the state with more favorable trends than the median had a never-burned refugium and/or infrequent fire management. They also all had substantial amounts of grazing, haying, and/or mowing managements. Sites with trends below the regional median trend had frequent or moderate fire management, and either a diminishing never-burned refugium or none at all. Regal populations at sites with ≤15 ha of grassland have become undetectable. Nonetheless, Hogback, a slightly larger than 15 ha site, had the most favorable trend, a significant increase. Nearly all Wisconsin Regal populations known before 1990 declined to consistent non-findability, even though these were conserved sites. More favorable trends at more recently discovered populations may be attributable to species-specific habitat management protocols implemented in the 1990s. Two sites with better than median long-term trends represent the longest consistent land ownership of known Regal populations in the state. This wide range of population outcomes illustrates both the need for long-term monitoring and the challenges of explaining the outcomes. Despite evidence of increasing Regal dispersal, this species remains very localized, indicating the unsuitability of the wider landscape as regal habitat. The number of significantly declining or no longer detectable populations in Wisconsin indicates an ever more adverse landscape for this species. Sites will need to have habitat characteristics that are ever more optimal in a wide range of climatic conditions for Regal populations to persist.

## 1. Introduction

Surveying and monitoring are necessary components of conservation programs for rare or declining butterfly species [1,2]. However, determining a butterfly’s status and trend is greatly complicated by large variation in abundance among generations attributable to climatic fluctuations, land use, and management activities, and sometimes other factors, such as disease, parasitoids, and density-dependent population regulation [3,4,5,6,7]. Our long-term study here exemplifies both the value of long-term monitoring data and the challenges of interpreting them.

Our study species is the regal fritillary (*Speyeria idalia*) (Lepidoptera: Nymphalidae), also referred to as “regal” or “regals” here. This butterfly is listed under state regulations as endangered in Wisconsin [8]. It is categorized as threatened, endangered, or extirpated in various other states and Canadian provinces, and is currently under review by the U.S. Fish and Wildlife Service for federal listing [9].

The regal fritillary primarily inhabits prairie, a native grassland in central North America [10,11,12]. Both within and outside the prairie region, localized populations have occurred in some old fields, damp meadows, and upland pastures, not necessarily native vegetation types [11,12,13,14,15,16]. Due to the vast destruction of prairie, and other grasslands, in the past two centuries, mostly for conversion to intensive agriculture, the regal fritillary has experienced widespread decline and range contraction. This is especially so in its eastern range, both in prairie (e.g., Illinois, Indiana) and east of prairie [10,14,17,18,19,20,21,22,23,24,25,26,27,28,29,30]. Much survey work has been conducted to assess this species’ status and trend in parts of its range [5,14,15,26,28,29,31,32,33,34,35,36]. Analysis of continent-wide data from 1977 to 2014 in the volunteer 4th of July Butterfly Count Program also supports the range-wide scale of the decline and range contraction of this spectacular and popular butterfly [37].

Like other large fritillaries (*Speyeria*), the regal fritillary has a single annual adult generation (known as the “flight period”) broadly spanning summer [11,12,17,19,20,21,22,25,30]. *Speyeria* flight periods tend to be asymmetrical, with a long attenuation later in summer after peak numbers. Most eggs are laid in late summer, singly and apparently haphazardly near, but seldom on, violets (*Viola*) (Violaceae) [38], the only reported larval food plants for *Speyeria*. Large fritillary individuals may disperse many kilometers, either to nectar or lay eggs. However, they are not migratory in the sense of regular movements between separate breeding and wintering grounds. Instead, *Speyeria* species overwinter within their year-round residential range as unfed first-instar larvae. In the laboratory, many *Speyeria* species can complete larval development using a variety of violet species.

The goals of our long-term study include the following: (1) follow-up on the status of historical localities if sufficient current observations by others were not available; (2) search for additional populations in potential habitat, especially at conserved sites because of the likelihood of long-term habitat security there; (3) use our, and others’, observations to increase the accuracy and specificity of the definition of high-potential habitat for this species, to improve efficiency of searches for new populations; (4) compile information necessary for designing and interpreting surveys to monitor regal populations (e.g., annual variation in flight period timing and relative abundance); (5) monitor individual populations in consecutive annual surveys, on a scale sufficient to distinguish population trends from annual fluctuations; (6) identify places of high priority for more follow-up on current status; and (7) note habitat and management factors associated with differences in relative population size and trend.

In this paper, we report data collected from 1988 through 2015 in Wisconsin [15,32,33,36]. We describe population trends over time at individual sites and pools of sites, and report site characteristics that are associated with population trends. Our results should be useful for evaluating Regal status and trends in Wisconsin, as well as devising monitoring protocols, interpreting survey data, and identifying what land management strategies occurred at sites with more favorable population trends. These findings should help in developing effective conservation strategies for this butterfly.

## 2. Methods

### 2.1. Study Sites

During summer 1988–1989, we made informal visits to familiarize ourselves with potential study sites and conducted preliminary surveys to develop the field methodology. We also began 12 years of studying regals in five other midwestern states to broaden our knowledge of the species’ habitat and management associations [39,40,41].

**Figure 1:**
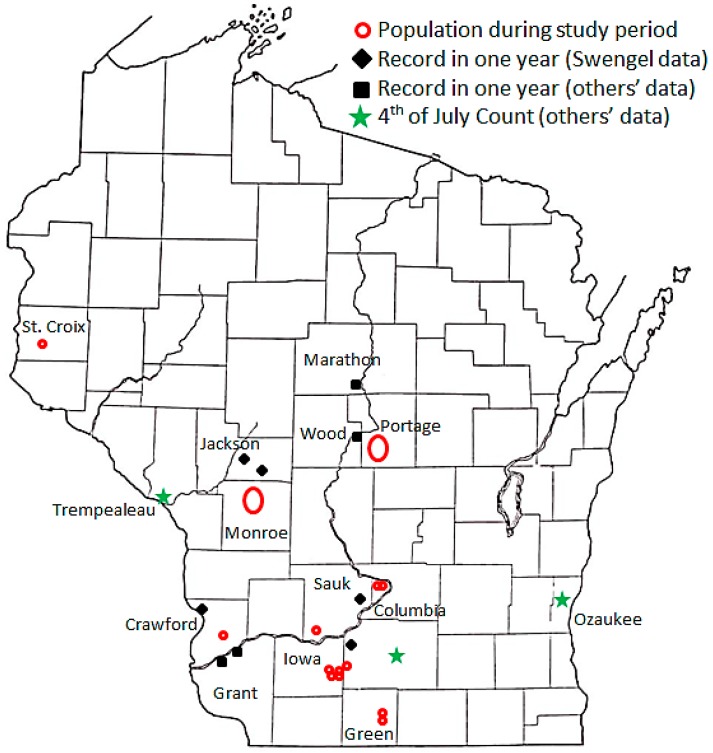
Map of Wisconsin. Counties are identified that contained a Regal fritillary population during the study period (1988–2015) and locations of one-year records on Swengel surveys, others’ observations, and/or a 4th of July Butterfly Count.

Starting in the 1990s, we formally surveyed six of 11 recent historic Wisconsin sites (records from 1970 to 1989), publicized our interest in others’ observations of this species (especially newly-discovered localities), and followed up on others’ reports of the regal fritillary as possible [32]. We visited each distinct area (“metapopulation”, defined loosely here as a site complex or cluster of sites) with a reported regal population (Figure 1), although we did not attempt to visit all known and potential sites within a site complex. At three additional historic sites, we visited only informally, as they did not appear to warrant formal survey effort due to degradation, urbanization, and/or very small size. We maintained a core group of sites surveyed each year once they were added to the study (Table 1; [33]).

In Southern Wisconsin, we surveyed 28 sites because they either had Regal records (historically or currently) or appeared plausible as potential habitat for this and/or other prairie-specialized species. We also added one site complex in central Wisconsin (Buena Vista Grassland, Portage County) in 1997 specifically to search for regal fritillaries, as well as study other grassland butterflies and birds. At many other survey sites in Central and Northern Wisconsin, we had other primary survey goals but our visits there during summer also afforded valid assays for the regal fritillary: (1) six state-owned wildlife areas containing complexes of old fields (degraded grasslands) to study grassland species, especially birds; most were in counties within the regal’s historic range [42,43,44]; (2) >125 pine-oak barrens (prairie-like herbaceous flora with trees and shrubs intermixed) in Central and Northwestern Wisconsin to study the Karner blue (*Lycaeides melissa samuelis*), federally listed as endangered [45], and other barrens butterflies [40,46,47]; and (3) >10 barrens and heaths across Northern Wisconsin (north of Karner blue range); and (4) 75 bogs and 20 bog roadsides in Northern Wisconsin to study their butterfly community, which includes other butterfly species that feed on violets as larvae [48,49,50]. Most sites were conserved lands in private or government ownership (e.g., preserves, federal refuges, state parks, state wildlife areas, state, and county forest) or public rights-of-way.

A single site of untilled grassland (in terms of contiguous tracts with the same ownership) was subdivided into multiple study sites if ≥200 m of wetland, woodland, intensive continuous farm grazing, and/or tilled land intervened among our sampling areas. However, we maintained separate time series for Thousand’s II and Thomson (subsequent acquisition) Prairies in Iowa County, even though they are about 100 m apart, because of their different survey histories (1990 on at the former, 1992 on at the latter). Buena Vista Grassland Wildlife Area in Portage County is a complex of eight sites (24–1350 ha) aggregated into one of the largest grassland complexes east of the Mississippi River, with about 5000 ha of public land and a large amount of surrounding private grassland [51].

Our long-term monitoring sites include all sites known in 1990–1992 to have Regal records during that period, as well as many of the Regal sites discovered subsequently. We discontinued consecutive-year surveying at Spring Green in 1999 because we had never found any regal fritillaries there; the last report was in 1990 [52,53]. However, we resumed surveying there in some years from 2005 to 2015, primarily for other target species. One population (in St. Croix County) was beyond the scope of our scheduling constraints to continue visiting after 2000. We surveyed here during 1998–2000 (three regal individuals found in 1998, one in 1999, and zero in 2000).

### 2.2. Butterfly Surveys

We conducted butterfly transect surveys along similar routes within each site each visit, similar to Pollard [54], as described in Swengel [39,40] and Swengel and Swengel [13,15,32,33]. We counted all adult butterflies observed ahead and to the sides, to the limit of species identification (possibly with binoculars after detection) and our ability to track individuals. We walked at a slow pace (1.5–2 km/h) on parallel routes 5–10 m apart, or down the middle of this route corridor if surveying alone. We tried to have both of us surveying together as much as possible for methodological consistency. Surveys by one person were conducted, however, to fill data gaps caused by scheduling constraints and poor weather on days when both surveyors could be fielded (Table 1). All surveys in this study were conducted by one or both of the authors, which may afford more methodological consistency than if all surveys were conducted by two people, but with variation in personnel.

We designated a new sampling unit whenever the vegetation along the route varied by management, type (wetland, wet, wet-mesic, mesic, dry-mesic, dry, “extra” dry sand), vegetative quality (amount of brush and diversity and abundance of native and exotic flora), and/or canopy. Starting in 2000, we also directly estimated percent shrub cover per unit. Routes crossed rather than followed ecotones and management boundaries to reduce edge effects, and were designed to minimize number of unit changes during the survey while covering representative areas of the site. Survey distance was approximately similar within unit among survey dates, and was measured based on topographic maps, landmarks, and property markers aligned to section (square mile) lines. At Buena Vista it was feasible to standardize the survey route to an 800 m length, typically a square (200 m per side) set in the center of a 40 acre (16.2 ha) square block of land. Some units had to be divided in half (two 400 m transects) when a management treatment occurred in only half the unit. At all other sites analyzed in this study, unit size and route length varied due to vegetative characteristics and size of land management treatments. For each unit survey, we recorded temperature and time spent surveying, and we estimated wind speed, percent time the sun was shining, and percent cloud cover. We surveyed in a wide range of times of day and weather, occasionally in intermittent light drizzle, if butterfly activity was apparent, but not in continuous rain. We kept data separate by unit survey.

In the first year or two of surveying at a site, the route within a unit or number of units surveyed might have been notably smaller than once we standardized the route for all subsequent long-term monitoring surveys. This initial pilot period occurred at Muralt Bluff (1988–1989), Spring Green (1989), and Pine Island site 1 (dog training area) in 1993–1994, as described previously [33]. After this initial pilot period, when we added units, we maintained them as separate time series. For example, we maintained Pine Island site 4 as a separate time series even though this area is adjoining to the east of site 1, the dog training area.

A unit’s management was coded based on treatments observed or evident during the study, including combinations (e.g., burning + mowing) as appropriate and following information available from the agencies that owned and/or managed the sites [32,33]. Fires typically occurred in a rotation of units burned in different years, possibly with some mowing or brush-cutting by hand or machine too. Non-broadcast managements (e.g., hand-cutting of brush) were counted as a treatment only in years when substantial alteration of vegetation occurred. Sites with no active broadcast management conducted or otherwise evident were categorized as “idle” (long-term unmanaged). In the early years of this study, Hogback was a non-conserved site with continuous moderate dairy grazing but cattle grazing did not occur after 1997, following conservation acquisition [33,55]. Cattle grazing (sometimes with horses, also) at Buena Vista was growing-season long, usually for one or two years at a time [15]. We observed cattle grazing at one other regal site (Thomson subsequent, 2012–2014).

Throughout the study, we conducted surveys on multiple dates each year at sites with Regal populations. We started no later than late June (except in the very cool growing season of 2004, when we began on 2 July) and continuing through July, to ensure we did not miss the onset of the regal’s main flight period. We were also attentive each year in the regal’s historic Wisconsin range to adults of the Aphrodite fritillary (*S. aphrodite*), a more widespread congenor similar but slightly earlier in flight period timing [33,56]. We also noted other butterfly species that emerge shortly before the regal fritillary as cues to check regal sites.

We required no ethical approval or research permits for this study because we did not handle or experiment on any animals and our survey sites were either open to public visitation or we obtained landowner permission to visit them.

### 2.3. Data Analysis

All statistics were calculated using ABstat 7.20 [57], with statistical significance set at two-tailed *p* < 0.05. Since we obtained significant results at a frequency well above expected from spurious Type I statistical error, we did not lower the critical *p* value further, as more Type II errors (biologically meaningful, but not statistically significant, patterns) would be generated than Type I errors eliminated. All statistical tests in this study are non-parametric, which do not require any assumptions about how the data are distributed (e.g., normality). All correlations were done with the Spearman rank correlation. To test for significant differences between categories, we used the Mann-Whitney U test.

To compare relative abundance, we calculated observation rates as total individuals per total survey distance per site (sum of units), using the peak survey at the site per year. Using the one peak survey during the main flight period avoids pseudoreplication (counting the same individual in more than one value in the dependent variable) and has been adequate for producing representative indices for comparisons of relative abundance within and among sites [35,47,58]. We calculated the median observation rate per year for pools of sites surveyed each year during 1990–2015 (*N* = 4 sites), 1992–2015 (*N* = 5 sites), and 1997–2015 (*N* = 7 sites) to serve as regional population abundance indices. At the scales of the site, site complex, and pools of sites, we correlated relative abundance with year to calculate trend. We calculated the median observation rate per year for pools of sites surveyed each year.

At Buena Vista Grassland, adequate samples were available to compare different methods of calculating abundance. On the scale of this entire site complex, we calculated relative abundance indices for mutually exclusive sets of long-term monitoring units: 1997–2015 (*N* = 12) and 2000–2015, which includes units surveyed 1998–2015 (*N* = 4), 1999–2015 (*N* = 2) and 2000–2015 (*N* = 4, with one unit not surveyed in 2006). We used the peak survey per unit per year, summed across all units in the set, then divided by the sum of survey distance. Another measure of relative abundance was based on the sum of all Regal individuals recorded in all unit surveys of all units, including re-surveys of the same unit and surveys of units done in only one or a few years. We divided this by the sum of survey distance, by year. We also calculated percent presence in all units surveyed during the flight period each year. Thus, we constructed four time series at the scale of the site complex: two mutually exclusive sets of long-term monitoring units, relative abundance in all unit surveys per year, and percent presence in all units surveyed each year. We compared these four different time series to each other in pair-wise correlations. The relative abundance time series all contained mutually exclusive data except for the series using all individuals from all surveys. For the latter, we subtracted out the regal individuals and survey distance represented in the other abundance time series in the correlation.

For all long-term monitoring units surveyed each year at Buena Vista Grassland during the period 1998–2015, we assembled time series of abundance in the long-term units subdivided geographically: North (*N* = 4 units, with one unit not surveyed in 2006), West Central (*N* = 8 units), Southeast (*N* = 2 units), and Southwest (*N* = 2 units). We correlated these four geographically segregated time series with year (trend) and in pair-wise correlations with each other. The spatial range of units surveyed in 1997 was not sufficient to include that year in this analysis by geography.

It was not valid to make one- and two-surveyor counts comparable by multiplying one-surveyor counts by two. On two-surveyor counts, we surveyed together in one party as done in the Christmas Bird Count and 4th of July Butterfly Count [59], and had one recorder to eliminate double-counting when both surveyors observed the same butterfly. Two surveyors would not necessarily record twice as many butterflies as one surveyor. It was also undesirable to limit analyses only to two-surveyor peak counts, as some of these were lower than one-surveyor counts at some sites in some years. It is not possible to know how many butterflies would have been recorded if both of us had been present for those one-surveyor counts. In prior analyses, the number of surveyors did not appear to have significant impacts on the statistical outcomes [15,33]. As a result, we included one-surveyor surveys in analysis without making any adjustments for comparability to two-surveyor surveys.

Instead, we disclose here which sites had how many one-surveyor surveys (Table 1). All long-term sites, except Pine Island, were primarily surveyed with two surveyors. They also had a two-surveyor count in years when the peak came from a count by one surveyor, except for most sites with one surveyor in 2012 and all sites with one surveyor 2013–2015. Muralt Bluff, Oliver, and the Thomson complex were primarily surveyed by two surveyors until 2012–2015. However, regal fritillaries at Muralt Bluff and Oliver had appeared to crash prior to 2012. Thus, comparing abundances from more recent one-surveyor results to prior two-surveyor results would likely have the most potential effect on trend analyses at the Thomson complex and Barneveld. Most surveys at Pine Island were done by one surveyor. The other sites primarily covered by one surveyor were either sites more recently added to the study or marginal for the regal fritillary with few records since 1990. Since comparability among sites in abundance would be also be affected by number of surveyors, we did no tests of differences in abundance among site complexes.

Starting in 2012, we participated in a single survey day per year at Fort McCoy, a large military reservation with >24,000 ha in Monroe County [60]. The target species was another prairie specialist butterfly, but the timing occurred during the main regal flight period. Due to safety and access issues, at least one staff biologist accompanied us, as well as other agency staff and/or volunteers. We recorded all butterfly observations by all observers in a unit as a single total of observations and survey effort per unit. Due to variation in units and routes within units surveyed per year, we calculated a single population index per survey day as total regal individuals per estimated total distance surveyed by the group (not distance walked by each individual surveyor). This method of calculating survey effort (by group or “party” surveying together, rather than by individual surveyor) is comparable to methods of the 4th of July Butterfly Count Program [59]. Due to the roughness of the data and few years from Fort McCoy, we provide summary statistics but no statistical analysis of this dataset.

## 3. Results

### 3.1. Patterns of Occurrence

**Figure 2:**
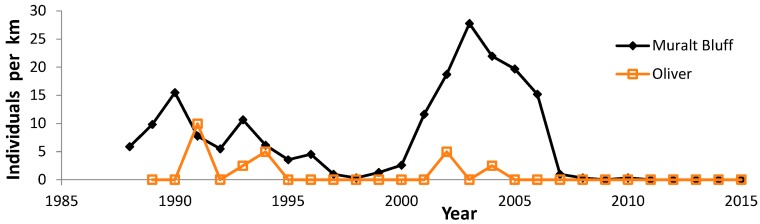
Regal fritillary individuals per km on peak survey per year at Muralt Bluff and Oliver Prairies in Green County.

**Figure 3:**
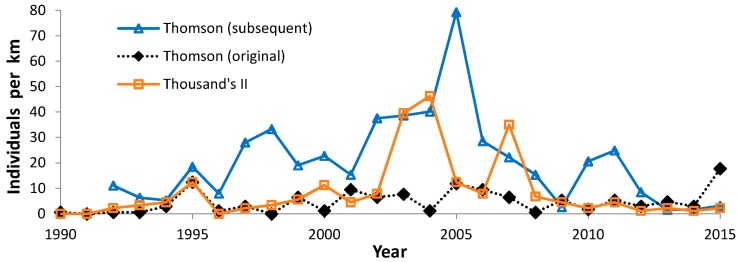
Regal fritillary individuals per km on peak survey per year at the Thomson Prairie Complex: Thomson (original acquisition), Thomson (subsequent acquisition), and Thousand’s II.

**Figure 4:**
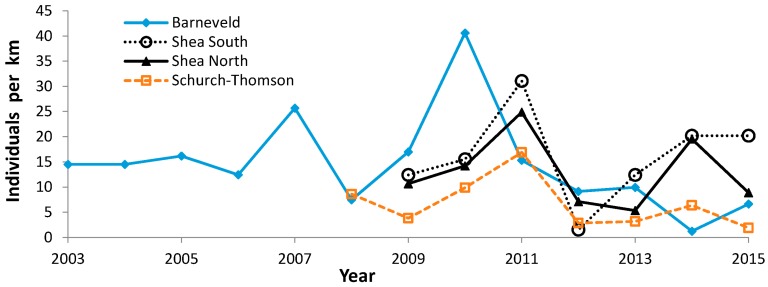
Regal fritillary individuals per km on peak survey per year at Barneveld, Shea (North, South), and Schurch-Thomson in Iowa County.

**Figure 5:**
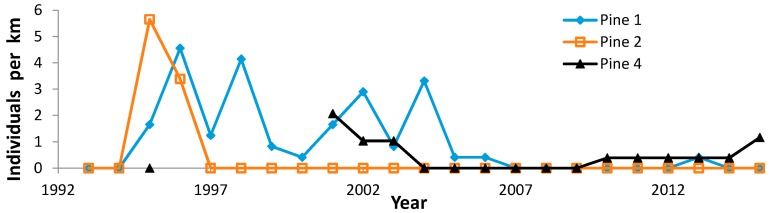
Regal fritillary individuals per km on peak survey per year at Pine Island 1 (dog training area), 2 (west), and 4 (east of dog training area).

**Figure 6:**
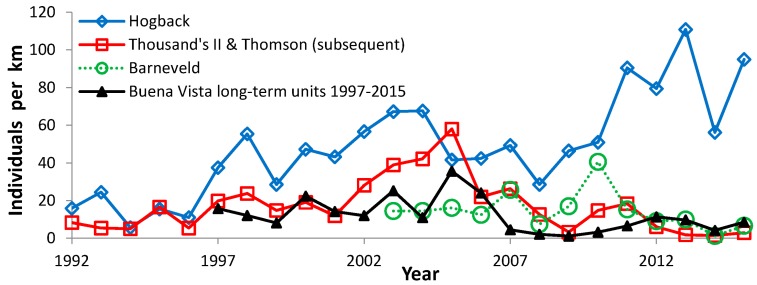
Regal fritillary individuals per km on peak survey per year at Hogback, Thousand’s II/Thomson (subsequent) combined, Barneveld, and Buena Vista (long-term units 1997–2015).

**Figure 7:**
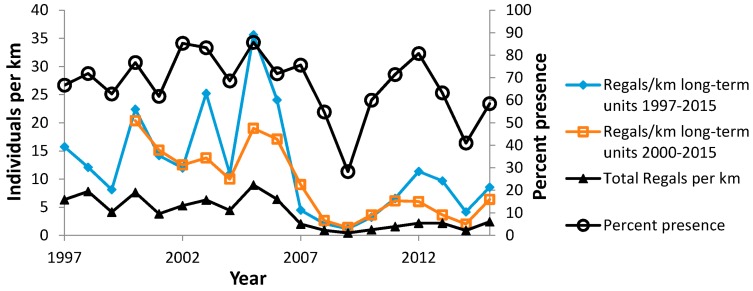
Regal fritillary incidence and abundance at Buena Vista Grassland: individuals per km on peak survey per year in units surveyed 1997–2015 and in the rest of the long-term units surveyed 2000–2015, and total individuals per total km surveyed each year (left axis), and percent presence in all units surveyed each year (right axis).

**Figure 8:**
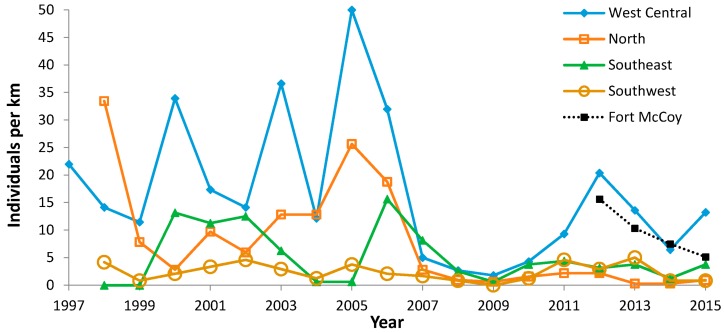
Regal fritillary individuals per km on peak survey per year at four areas of Buena Vista Grassland (West Central, North, Southeast, Southwest) and Fort McCoy. The 2016 abundance index at Fort McCoy was 16.2.

During 1988–2015, we recorded 5609 Regal fritillaries in 442.7 km on the peak survey per year at populations (sites where we recorded the species in more than one year) in southern Wisconsin (Figure 2, Figure 3, Figure 4 and Figure 5, with sites grouped geographically; Figure 6). At Buena Vista Grassland in central Wisconsin (Figure 6, Figure 7 and Figure 8), we re-surveyed throughout the flight period. We recorded 3428 regal fritillaries in 300 km on the peak survey in 22 units surveyed from 1997 to 2000 onward (one unit in the 2000–2015 set not surveyed in 2006), and a total of 4040 individuals in 1086 km in all surveys of all units during the 1997–2015 flight periods. We recorded any Regal fritillaries on at least one survey of 98 units spread across the entire east–west (12.5 km) and north–south (16.5 km) breadth of the Buena Vista site complex, and did not ever record the species in the remaining 20 units surveyed at least once during the flight period. At Fort McCoy (Figure 8), where the survey method varied more than elsewhere among years, we and 3–4 other observers per year recorded 339 individuals in 39.4 km during 2012–2015.

We also surveyed other prairie and grassland sites both in the known current range (in, and south of, counties with populations as mapped in Figure 1) and north of that range in Wisconsin. At a number of sites with plausible habitat, we did not find any regal fritillaries and know of no such records by anyone else (Appendix A Table A1).

**Figure 9:**
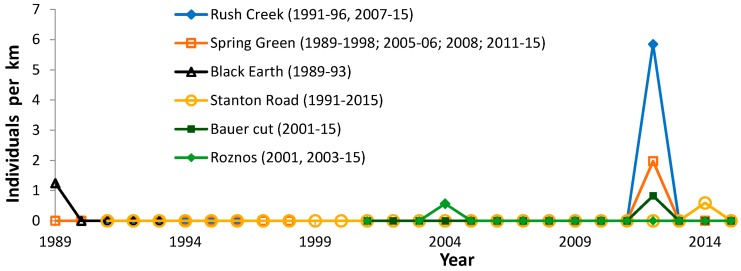
Regal fritillary individuals per km on peak survey per year at six sites with a record in only one year in this study. This represents 13 Regal fritillary individuals in a total of 169.3 km of surveying on the “peak” survey per year. Roznos Meadows in Devil’s Lake State Park is also reported in the Baraboo 4th of July Butterfly Count and Stanton Road is reported in the Wazee 4th of July Butterfly Count (Table 4).

At six sites surveyed for at least five years (14–25 years at five sites), we found any regal fritillaries in only one year (Figure 9). Although one of these records occurred in 1989, they are skewed to the 21st century (five of six sites) and later in the time series (the last 20% of the time series at 4/6 sites). Half of these records occurred in 2012, the year with the earliest (warmest) seasonal development in this study as evidenced by flight period data (Table 2). A few other sporadic records have also been reported by volunteers in the last decade (Table 3).

The only records for the regal fritillary in Wisconsin in the 4th of July Butterfly Count Program are from five count circles, and only in one year per circle (Table 4). These records are similarly biased later in the time series: 4/5 are in the latter 50% of the published time series (2/3 when excluding the Baraboo and Wazee Counts, which are also represented in Figure 9).

### 3.2. Flight Period Characteristics

At Buena Vista, we have the most complete record of flight period data (Table 2). Peak timing in the long-term units varied among years by 33 days (22 June–25 July) for the mean peak date and 32 days (17 June–19 July) for the median. Within year, mean and median peak date varied by 0–7 days. However, at some units, the peak occurred much later (in August).

Starting in 2006, we surveyed at Buena Vista both shortly before and shortly after the flight period, so that the entire flight period was more precisely documented. In that period, the first date and mean peak date correlated positively and significantly (*r* = 0.83891, *N* = 10, *p* < 0.01), as expected due to seasonal phenology. However, the flight period span and the span when peak dates occurred each year did not correlate significantly (*r* = 0.23780, *N* = 10, *p* > 0.10), so that the period of peak numbers did not lengthen or shorten significantly in relation to flight period length. However, the flight period span did correlate negatively and significantly with mean peak date each year. (*r* = −0.82675, *N* = 10, *p* < 0.01), so that earlier peak dates occurred in years with longer flight periods. The flight period span also correlated positively and significantly with regal abundance as measured at all the long-term monitoring units (surveyed from 1997 to 2000 on) (*r* = 0.64438, *N* = 10, *p* < 0.05). Thus, longer flight periods also occurred when regal fritillaries were more abundant. Throughout the entire study here (1997–2015), mean peak date did not correlate significantly with any measure of Regal abundance at the scale of this site complex (pair-wise correlations of mean peak date with total regals per total km, percent presence in units, and abundance on the 1997–2015 long-term units, *N* = 19, *p* > 0.10 for each). As a result, both regal abundance and seasonal phenology appear to be independent influences on flight period span.

### 3.3. Population Trends

The populations in our long-term monitoring sites varied greatly in trend over time (Table 5). Hogback had the most favorable trend (significant increase), both in comparison of trends calculated with all years of data available and when limited to 1997–2015 to control for time period among sites.

The poorest outcome occurred at formerly reliable populations that declined to consistent non-detectability (Figure 2). At Muralt Bluff, we found none in the last five years, and only a single individual once during 2009–2015 (in 2010). Oliver was reported to have reliable regal detection prior to this study [53] but we only found inconsistent regal occurrence, and none during 2005–2015. Muralt Bluff had a strongly significant negative trend but Oliver had only a non-significant decline because we detected so few individuals and so sporadically, although these were entirely in the first 3/5 of the study (Table 5). Spring Green formerly had a reliable population prior to this study [53]. However, with only one year of regal observation in our surveys (Figure 9), we did not calculate a trend for this site. We do not know whether Pine Island 2 (west) ever had a reliable population. We found the Regal fritillary there in only two years (1995–1996) during 1993–2015 (Figure 5). Pine Island 1 (dog training area) declined significantly to inconsistent detection recently. However, an area adjacent to this but not surveyed as many years (site 4, east) has continued to have reliable findability in recent years, but in low numbers (Figure 5).

Since regal fritillaries occurred throughout the very large area of the Buena Vista Grassland site complex, we used several strategies for assessing occurrence and abundance. On the scale of this entire site complex (Figure 7), a variety of independent measures of regal presence and abundance covaried very strongly (Table 6). However, when the long-term monitoring units at Buena Vista were grouped geographically into four areas, only two of six pair-wise correlations of abundance were significant, although all were positive (Table 7). These different areas of Buena Vista had different long-term trends (Table 8), from no trend (Southeast) to mild non-significant negative trends (West Central, Southwest), to significant decrease (North). Regal abundance was significantly highest in the West Central area, while the other areas did not differ significantly from each other in abundance (Table 9, Figure 8).

This variation in trend among sites in a complex is also apparent at the Thomson complex. Thomson (original) has increased (significantly so over the entire study period), while Thousand’s II and Thomson (subsequent) had less favorable trends (Table 5): a non-significant positive trend at the former and a negative trend (significantly so during 1997–2015) at the latter. It remains possible that the four recent years of one-surveyor surveys here made the calculation of long-term trend less favorable than it would have been with two-surveyor surveys. Thus, these results should be viewed as rougher than at the other sites. But our highest regal count ever in the 26-year time series at Thomson (original) was by one surveyor in 2015 (Figure 3).

Fort McCoy data are rougher due to variation among years in number and identity of surveyors and in locations surveyed at this large site complex. The relative abundance and direction of change in abundance among years were generally consistent with results at Buena Vista, also in Central Wisconsin (Figure 8). The abundance index in 2016 was 16.2 (the highest index in this time series), when one staff biologist and no one else surveyed with us. The high 2016 abundance indicates there was not a consistent decline at this site in the last five years.

### 3.4. Comparisons of Site Characteristics

**Figure 10:**
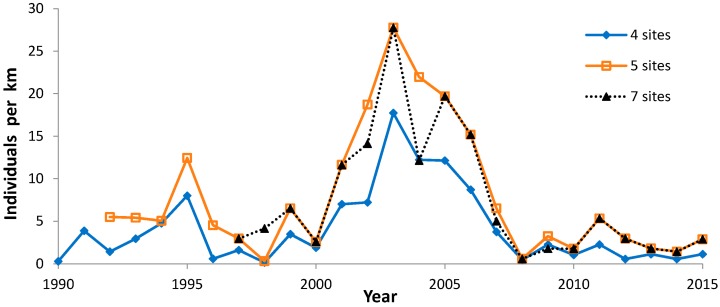
Median regal fritillary individuals per km on peak survey per year at sites surveyed each year: four sites surveyed 1990–2015 (Muralt Bluff, Oliver, Thomson original, Thousand’s II), five sites surveyed 1992–2015 (Muralt Bluff, Oliver, Thousand’s II/Thomson subsequent combined, Thomson (original), Hogback), and seven sites surveyed 1997–2015 (the five sites plus Pine Island 1 and Buena Vista West Central areas).

We identified the median abundance index per year for sites surveyed each year in the period (Figure 10). This median abundance was low early in the 1990s, peaked in the middle of the study, and was persistently low again in recent years (Figure 10). The two longest running of these median time series had mild non-significant negative trends (Table 10). The shortest series began in the period of peak abundance and had a significant negative trend (Table 10).

We ordinated sites by trend (increasing, relatively stable, decreasing) and classified them by site factors (patch size, fire and other management activities) for the seven long-term monitoring sites surveyed 1997–2015 (Table 11). Four sites had a more favorable trend than the regional median trend (Table 10). But one of these (Oliver) had 11 years of a zero abundance index at the end of its time series. As a result, Oliver had the negative outcome of appearing no longer to support a reliably detectable population. The three other populations had a never-burned refugium and/or infrequent fire either throughout or later in the study period. They also all had substantial amounts of alternative managements (e.g., grazing, haying, mowing). While Thomson (original) had frequent fire (with 100% fire) early in the study, this was mitigated by being embedded in a site complex that was not 100% burned at once. This site also had large amounts of mowing management throughout the study, and very little fire in the second half of the study. The sites with poorer trends than the median had either frequent or moderate fire. They also had either a diminishing never-burned refugium or none at all. All of the smallest sites (15 ha of grassland or less, with no plantings either) have become undetectable recently. Nonetheless, Hogback was just slightly larger than 15 ha and has had the most favorable positive trend.

## 4. Discussion

### 4.1. Patterns of Occurrence

On the one hand, many sources of data (Table 3 and Table 4, Appendix A Table A1) indicate the overall rarity and localization of the regal fritillary in Wisconsin (Figure 1) and range-wide [37]. An observer must visit relatively few and very particular sites to find this butterfly reliably, which rarely turns up elsewhere in the landscape.

On the other hand, there is some evidence that regal fritillaries occasionally disperse out from a population. Regal occurrence in only one of many years of our surveys at a site (Figure 9) is consistent with similar single-date or single-year records for an area reported by others in the 4th of July Butterfly Count Program (Table 4) and other volunteer data (Table 3). The clustering of most of these sporadic records in and adjoining counties with regal populations (Figure 1) supports that these sporadic records may be symptomatic of dispersal from a relatively near population. In addition, proximity of some sporadic records to riverways (as evident by serpentine county and state borders in Figure 1) suggests that these topographic features may serve as dispersal corridors.

These sporadic records appear to have increased in Wisconsin in recent years, both in our surveys (Figure 9) and in the 4th of July Count Program (Table 4). In both these data sources, effort is accounted for (i.e., years with zero observations are documented). Thus, this increase in sporadic records appears to be a true pattern and not just a consequence of increased survey effort (more observers and/or more field days) and/or increased interest in reporting the species. Since Wisconsin is at the northern edge of the species’ range, this may be a possible consequence of climate change. Many butterfly species have been documented to increase in abundance in their northern range, and expand their range, in recent decades in association with warming climate [61,62,63,64,65,66].

While these sporadic records indicate the potential for regal fritillaries to disperse out of localized population areas, they also indicate very limited effectiveness of this dispersal to found reliably detectable populations afterwards. This assessment is based on well surveyed sites rarely having a consistently detectable regal population discovered in them many years after consistent surveying would have been adequate for finding regal fritillaries in them (Figure 9 and Appendix A Table A1).

Fort McCoy is a probable exception. Either the regal fritillary colonized at this northern range edge recently, or a population occurred here all along but was undetected despite a number of knowledgeable surveyors on the site for many years. Some of those prior surveys included specific searches for this species [67], while others were targeting summer Karner blues [68,69,70] or grassland birds [60] in areas that now have regal records. Although the data in 2012–2015 suggest a decline at Fort McCoy (Figure 8), the population index in 2016 was just above the highest value in 2012–2015, suggesting overall stability in this rough dataset. The much more noteworthy result is that the regal fritillary has reliably occurred here each year since being discovered in July 2010 (Tim Wilder, pers. comm.; Figure 8). Not graphed are the years of non-detection in prior years. Thus, any positive population index now represents an increase over the prior situation of not being discovered.

This outcome at Fort McCoy fits an expectation of increasing abundance and/or range expansion at a species’ northern range margin because of warming climate [61,62,64,66]. However, Fort McCoy also fits the definition of high-quality regal habitat as defined by midwestern regal populations: large untilled grassland with a mix of both uplands and swales and unintensive land uses compatible with maintaining both the native flora and the butterflies themselves in all their life stages [5,13,31,34,38]. While fire occurs at Fort McCoy, the fire return interval varies greatly, from annually in weapon firing zones, to frequent, moderate, infrequent, and never (as defined in Table 11). Thus, increased observation of the regal fritillary at Fort McCoy supports that suitable habitat conditions are essential for butterflies to persist successfully in the landscape [65,71,72,73,74,75].

These complex messages of extreme rarity and localization contrasting with dispersal and colonization have also been documented elsewhere. In isolated Iowa prairies, regal fritillaries overwhelmingly turned back at habitat margins instead of dispersing out of the site into non-habitat [76]. The regal fritillary has also been documented as localized and declining in Illinois and Indiana [77]. However, this butterfly has recently and unexpectedly expanded in the highly-developed landscape of Northeastern Illinois and Northwestern Indiana recently [77]. In a large-scale conservation effort there, tilled fields around remnant prairies were acquired for re-vegetation to native prairie flora. The two violet species deliberately included in the diverse plantings established only sparsely. However, a ruderal annual violet unexpectedly established profusely in both the plantings and in unplanted old fields that developed once tilling ceased. Regal fritillaries have recently increased and expanded within the project area outside the prairie remnants, and achieved equal abundances in prairie plantings and old fields. This butterfly also dispersed and colonized on a landscape scale of 5–10 km and more to re-occupy historic sites as well as colonize other sites. On the one hand, these results support that the regal fritillary is usually localized because of the paucity of suitable vegetation in compatible land uses and managements, but when required floristic resources consistently abound on a landscape scale, the regal fritillary is able to respond and colonize suitable habitat patches. 

### 4.2. Flight Period Characteristics

At Buena Vista, the flight span was longer when peak dates were earlier—i.e., in warmer years (Table 2). Thus, survey timing must not only be shifted earlier or later due to climatic variation, but the length of the survey period also varies from year to year. Independent of that, flight period span also correlated positively with abundance. Thus, phenology is not the only factor determining how long the flight period (and survey season) is in a year. More analysis is needed to develop predictions of regal abundance in the next flight period in relation to observed climate conditions during the immature life stages. Until then, it is easier to anticipate when the peak flight period will occur, based on seasonal development and first observed dates, than how long the flight will last in the summer based on anticipated regal abundance (e.g., for planning personnel schedules for presence/absence surveying).

### 4.3. Population Trends

Median abundance (Figure 10) is useful for comparing which sites had more or less favorable trends than the median regional value (Table 5 and Table 11). Collectively, these population values exhibited high interannual variability, with highest abundance in the middle of the study (early 2000s). Most sites individually also exhibited a very wide range of abundance as well (Figure 2: Muralt Bluff; Figure 3, Figure 4, and Figure 6, Figure 7 and Figure 8). This suggests that regional climatic variation has been one important determinant of regal abundance in Wisconsin.

However, site-specific factors of habitat and land use are also strongly implicated in regal population outcomes. For example, a few populations had consistently lower population values throughout the study period, in contrast to relatively high median abundance values (Figure 10). This includes Pine Island (Figure 5), a large site complex but containing degraded old field (Table 11), and Oliver (Figure 2), a high-quality prairie but very small and near Muralt Bluff, which also was also relatively small (Table 11). Thus, these sites did not register higher abundance values that appear attributable to regional climatic patterns.

Furthermore, nearly all Wisconsin regal populations known before 1990 declined to apparently consistent non-findability of the populations: Muralt Bluff and Oliver (Figure 2), and Spring Green (Figure 9). The exception is one low-density population with regal fritillaries still recorded as present at the end of this study: Pine Island site 1 dog training area and site 4 east of this (Figure 5). Spring Green’s population was last seen in 1990 [53]. Then individuals were next observed here in 2012 by multiple observers (Figure 9; Table 3), but not in following years. This suggests that 2012 observations here represented transient dispersal from somewhere else rather than re-detection or re-establishment of this population. Thus, even though these historical populations were in conserved sites, which did not result in a beneficial long-term outcome for regal populations at most of these sites.

With increased concern about the regal fritillary and more search effort by many observers, as summarized in [32], more populations were discovered in the 1990s. This included Hogback, Thomson complex and additional areas in Iowa County in the vicinity of Thomson, and Buena Vista Grassland (Figure 3, Figure 4, and Figure 6, Figure 7 and Figure 8) [32,33,78].

Swengel et al. [36] reported 3/8 positive trends to any degree and 5/8 negative to any degree for Wisconsin regal populations, which was not a significant skewing of trend in one direction or the other. Since that analysis, the extant populations have exhibited a wide range of population trends (Table 5), including a number of significant declines. By contrast, Hogback has the most favorable trend (significant increase). Fort McCoy has also increased from consistent non-observation to consistent detection. Two portions of the Thomson complex also have positive trends (significant at original, non-significant at Thousand’s II), while the third portion has a significant decline. Buena Vista also has variability in trend among different areas within this site complex (Table 7), including apparent stability. At this last site, the lack of positive trends (compared to some Southern Wisconsin sites) may be due to the lack of data from earlier in the 1990s. The Buena Vista time series started right before or during the peak period of median abundance for Wisconsin populations collectively (Figure 10).

In the 1990s, Wisconsin implemented species-specific habitat management protocols for rare butterfly conservation [36,55]. The regal fritillary protocol specified allowable fire regimes and encouraged mowing, permanent non-fire refugia, and monitoring the butterfly. This appeared to confer benefit on regal trend, compared to generalized ecosystem or habitat conservation approaches in Iowa and historic Wisconsin regal sites from the 1970s and 1980s. Whether these protocols are sufficient, and sufficiently complied with, to maintain regal populations long-term at these more recently discovered sites remains to be seen in the coming decades. That is, has the overwhelming decline for the populations known in the 1970s and 1980s been adequately turned around at sites discovered since?

### 4.4. Comparisons of Site Characteristics

Regal fritillary trends are a combination of regional climatic and site-specific habitat factors including vegetative composition, land use/management, and the landscape context [13,15,26,33,34,55,79]. Additionally, isolated population pathology may be an influence, as well as greater variability at range edge [80,81]. Each population area is its own unique instance of site and landscape factors (Table 11) and climatic conditions. It is difficult to parse these factors statistically in long-term population time series because of the distance among population areas, leading to variation in climate. Thus, broad-scale surveying and analysis across many sites in many states have been invaluable in identifying factors relevant to regal incidence and abundance [5,26,34]. Habitat and land management factors have been analyzed extensively [13,15,16,31,39,40,41,55,76,79,81,82,83,84] but we are not aware of any analysis of regal data related to climatic variation in the Midwest or elsewhere.

A synergy (positive or negative) between length of land ownership is also evident in Wisconsin’s regal fritillary outcomes. On the one hand, there is a lag time of years or decades between when management implicated as adverse for the regal fritillary begins and when the regal population declines to non-findability (Table 11). On the other hand, Fort McCoy has an outstanding butterfly fauna besides regal fritillary [67,68,69,70], and represents the single longest landowner and land use of any known regal population in the state. Fire frequency varies greatly among parts of this military reservation, from frequent to moderate to infrequent to none very long-term. Other land use activities, both deliberately for vegetative management (brush cutting, timber sales, exotic plant control) and inadvertent consequences of military exercises (bivouacking, tank driving, and so on), also occur here. Vegetative structures vary from grassland (both short and taller turfs) to savanna to forest, all of these in varying patch sizes.

The second longest consistent ownership (>60 years) and land use approach for a Wisconsin regal population is Buena Vista Grassland, managed by the Wisconsin Department of Natural Resources for Greater Prairie-Chicken (*Tympanuchus cupido pinnatus*) [51]. Both of these site complexes hint that long-term consistency of compatible land uses in large patches of habitat is important for regal population persistence.

## 5. Conclusions

The wide range of population outcomes documented in this study illustrates both the need and the challenge of anticipating future effects of climatic variation and site-specific habitat characteristics and their landscape context. Our study provides evidence of increasing regal dispersal and northward population expansion, possibly in response to climate. Despite this, the regal fritillary remains very localized to known population areas, indicating the unsuitability of the wider landscape as regal habitat. Very large fluctuations in abundance exhibited by populations with stable or even increasing trends indicates that extreme weather can be adverse for regal abundance, even though this butterfly has a wide climatic tolerance based on its large range. The relatively large number of significantly declining or no longer detectable populations in Wisconsin indicates an ever more adverse landscape for this species. In such a circumstance, it is reasonable to expect that a site will need to have habitat characteristics that are ever more optimal, and optimal for a wide range of climatic conditions, for a regal population to persist viably.

## Figures and Tables

**Table 1:** Descriptive statistics for each site (county in parentheses) on the number of years during 1988–2015 peak counts were obtained by one surveyor or two surveyors. Before 1998, all peak counts by a single surveyor occurred in years with two surveyor visits during the flight period.

Site (County)	*N* Surveyors		Years of Peak Counts by *N* Surveyors	
	One	Two	One Surveyor	Two Surveyors
Long-Term Monitoring Sites				
Hogback (Crawford)	1	23	1995	
Muralt Bluff (Green)	6	22	1996, 2006, 2012–2015	
Oliver (Green)	5	21	1996, 2012–2015	
Pine Island (Columbia)				
site 1: dog training area	17	6		1996, 1998–2002
site 2: west	17	6		1996, 1998–2002
site 4: adjacent to, and east, of 1	15	1		2001
Thomson complex (Dane, Iowa)				
Thomson subsequent	5	19	1994, 2012–2015	
Thomson original	6	20	1994, 1996, 2012–2015	
Thousand’s II	5	21	1996, 2012–2015	
Buena Vista:				
West Central	0	19		
North	1	18	1 unit in 2012	
Southeast	1	18	2 units in 2012	
Southwest	0	18		
Other populations surveyed				
Barneveld (Iowa)	4	9	2012–2015	
Schurch-Thompson (Iowa)	7	1	2009	
Shea North (Iowa)	5	2	2009, 2011	
Shea South (Iowa)	5	2	2009, 2011	
Species found in only one year				
Bauer cut (Jackson)	0	15		
Stanton Road (Jackson)	2	23	1994, 2013	
Roznos Meadows (Sauk)	14	0		
Rush Creek (Crawford)	0	15		
Spring Green (Sauk)	6	18	2005–2006, 2011, 2013–2015	

**Table 2:** Descriptive statistics on regal fritillary flight period and peak dates at Buena Vista Grassland. Dates (MDD, or month-day) indicate last survey date before observed flight period, date of first and last observation, and first survey date after observed flight period. For the long-term monitoring units, dates are provided for the mean and median of the peak surveys, and the minimum (min, i.e., earliest) and maximum (max, i.e., latest) peak survey date.

Year	Last	MDD First	MDD Last	First	Flight Span	Peak Survey in Long-Term Monitoring Units				
	MDD			MDD		MDD	MDD	MDD	MDD	Days
	Before			After		Mean	Median	Min	Max	Range
1997	514	702	828		57	721	716	716	730	14
1998	603	625	826		62	706	701	624	826	62
1999	609	623	817		55	717	714	706	804	29
2000	622	628	822	1004	55	713	711	704	802	29
2001	529	625	915	1014	82	715	711	710	807	28
2002	622	629	901		64	713	711	705	803	29
2003	628	629	906	1004	69	722	718	705	906	63
2004	521	702	911		71	725	718	718	829	42
2005	615	702	910	1015	70	709	709	702	715	13
2006	615	630	826	909	57	710	708	630	722	22
2007	617	630	903	908	65	705	704	630	715	15
2008	706	712	816	822	35	722	719	712	802	21
2009	626	705	823	827	49	716	712	705	823	49
2010	612	616	822	827	67	709	709	625	723	28
2011	613	701	826	909	56	716	715	704	807	34
2012	608	615	825	922	71	622	617	615	720	35
2013	619	704	906	913	64	719	714	714	816	33
2014	614	628	824	906	57	713	710	704	730	26
2015	616	622	830	912	69	707	703	627	815	49

**Table 3:** Regal fritillary records reported directly to us or to a reporting website, representing sites without a known Regal population.

Date	Site	County	Source
4 July 2007	Woodman-Millville unit	Grant	Todd Sime (pers. comm. Karl Legler)
16 August 2011	Semrad Slough	Grant	Karl Legler (pers. comm. KL)
8 June 2012	Spring Green Preserve	Sauk	Ann Thering: www.wisconsinbutterflies.org
6 July 2012	Williams Street garden, Village of Biron	Wood	Connie Stout: www.wisconsinbutterflies.org
1 September 2013	Big Eau Pleine County Park	Marathon	Dan Belter: www.wisconsinbutterflies.org

**Table 4:** Regal fritillary reports in Wisconsin 4th of July Butterfly Counts. See Appendix B for report citations. Roznos Meadows in Devil’s Lake State Park is also reported in the Baraboo count and Stanton Road is reported in the Wazee count (Table 1 and Figure 9). Latitude and longitude are for the center of the 15-mile diameter count circle.

Count	Latitude	Longitude	*N* Years Held	*N* Years Regals Found	FirstYear Held	Last Year Held	Total Regal Individuals	Year Regal Found	*N*th Year Regal Found
Baraboo	43.48	−89.72	30	1	1986	2015	1	2004	19
Madison	43.1	−89.38	25	1	1991	2015	1	2004	14
Riveredge	43.4	−87.98	20	1	1987	2013	1	2003	12
Trempealeau	44.07	−91.40	19	1	1997	2015	2	1997	1
Wazee	44.28	−90.67	22	1	1994	2015	1	2014	21

**Table 5:** Spearman rank correlations of trend (abundance vs. year). These were calculated for populations (sites where regal fritillaries were recorded in more than one year in this study) surveyed >12 years, with county in parentheses. NS = not significant (*p* > 0.10). *p* < 0.10 is considered near significant.

Site (County)	All Years Available			1997–2015 (*N* = 19 Years)	
	*N*	*r*	*p*	*R*	*p*
Hogback (Crawford)	24	0.76321	<0.01	0.55024	<0.05
Muralt Bluff (Green)	28	−0.55477	<0.01	−0.6662	<0.01
Oliver (Green)	26	−0.38383	<0.10	−0.1909	NS
Pine Island (Columbia)					
site 1 (dog training area)	23	−0.526	0.01	−0.7971	<0.01
site 2 (west)	23	−0.39606	<0.10	incalculable (all zeroes)	
Thomson complex (Dane, Iowa)					
Thousand’s II	26	0.10565	NS	−0.4355	<0.10
Thomson-original	26	0.42929	<0.05	0.11547	NS
Thomson-subsequent	24	−0.17834	NS	−0.6058	<0.01
Thousand’s II & Thomson sub.	24	−0.19313	NS	−0.5994	<0.01
Buena Vista complex (Portage)					
units done 1997 to 2015	19	−0.54035	<0.05	−0.5404	<0.01
units done 1998–2000 to 2015	16	−0.74614	<0.01	--	
Percent presence	19	−0.37544	NS	−0.3754	NS
Total individuals/total km	19	−0.69298	<0.01	−0.693	<0.01
West Central area	19	−0.43879	<0.10	−0.4388	<0.10
Barneveld	13	−0.48143	<0.10	--	

**Table 6:** Pairwise Spearman rank correlations of different measures of Regal fritillary incidence and abundance at Buena Vista Grassland, at the scale of the entire site complex. In the correlations of relative abundance (RA) between long-term monitoring units and total individuals/total km, we excluded from this latter measure the data in the long-term time series it was being compared to.

Unit or Site Indices Being Correlated	*N* Years	*r*	*p*
RA on units surveyed each year 1997–2015 with:			
Percent presence in all units surveyed each year	19	0.71930	<0.001
RA on units only surveyed from 1998–2000 to 2015	16	0.89036	<0.001
RA of total individuals/total km	19	0.74561	<0.001
RA on units only surveyed each year 1998–2000 to 2015 with:			
Percent presence in all units surveyed each year	16	0.72701	<0.01
RA of total individuals/total km	16	0.90213	<0.001
Percent presence in all units surveyed each year with			
RA of total regals/total km (all data)	19	0.70000	<0.001

**Table 7:** Pairwise Spearman rank correlations to test for spatial synchrony in abundance in long-term monitoring units among subregions at Buena Vista Grassland during 1998–2015. *N* = 18 years for all correlations. NS = not significant (*p* > 0.10).

Subregion	North		Southeast		Southwest	
	*r*	*p*	*r*	*p*	*r*	*p*
West Central	+0.62837	<0.01	+0.32625	NS	+0.56944	<0.05
North			+0.02287	NS	+0.35644	NS
Southeast					+0.32951	NS

**Table 8:** Spearman rank correlations of trend (abundance vs. year) at Buena Vista Grassland by geography. This is for the period 1998–2015 (maximum number of years possible while keeping number of years constant among areas). NS = not significant (*p* > 0.10).

Subregion	*N* Years	*r*	*p*
West Central	18	−0.39339	NS
North	18	−0.76979	<0.01
Southeast	18	−0.00829	NS
Southwest	18	−0.20479	NS

**Table 9:** One-tailed *p* values from pairwise Mann-Whitney tests of difference in abundance in long-term monitoring units 1998–2015 between subregions at Buena Vista Grassland. *N* = 18 years for all tests. Significant values (*p* < 0.025) are shown in boldfaced italics.

Subregion	North	Southeast	Southwest
West Central	0.0045	0.0004	<0.0001
North		0.3462	0.0946
Southeast			0.1446

**Table 10:** Spearman rank correlations of trend (abundance vs. year) in median abundance of pools of sites monitored each year during the period (Figure 10). NS = not significant (*p* > 0.10).

Pool of Sites	*N* Years	*r*	*p*
Four sites 1990–2015	26	−0.14195	NS
Five sites 1992–2015	24	−0.30492	NS
Seven sites 1997–2015	19	−0.45722	<0.05

**Table 11:** Characteristics of sites analyzed for trend during 1997–2015 (Table 5). Sites are listed from most positive (+) to most negative (−) trend, with the trend of the median abundance for these sites (negative at *p* < 0.05, per Table 10) also indicated. The bottom site has no calculated trend because no regal fritillaries were recorded there during this period.

Site	*N* Zeroes at End of Time Series	Prairie ha	Grassland ha	Prairie Planting	Never Burned Refugium	Fire Return Interval in Burned Units	Maximum Fire Extent in a Year	Other Broadcast Management
+ *p* < 0.05								
Hogback	0	16 *	16 *	yes	Yes	Zero then Mod	30%	only CG until 1997; then Idle until 2004; then F and BC; also GG began in 2014.
+ *p* > 0.10								
Thomson original	0	5	121 **	no	No	Freq then Infreq	100%	Freq mow
− *p* > 0.10								
Oliver		2	2	no	no	Freq	100%	Negligible
− *p* < 0.10								
Buena Vista West Central	0	0	1350 ***	yes but tiny	yes	Infreq	<5%	Infreq but every year in parts: CG; CHG; Hay; Mow; Herb
− *p* < 0.05								
Median of 7 sites	0							
− *p* < 0.01								
Thousand’s II	0	2	121 **	yes	no	Freq then Mod	100%	Infreq mow
Thomson subsequent	0	32	121 **	yes	yes but decreasing to <0.5 ha	Mod	50%	Infreq mow and BC; CG 2012–2014 entire patch
Muralt Bluff	5	15	15	no	No ****	Freq	90%	Mod then freq BC + mow
Pine Island 1 (dog training)	2 but 0 in 2007–2012 also	20	121	no	no	Mod	50%	Infreq then mod BC and mow; recent herb
No trend								
Pine Island 2 (west)	19	12	12	no	no	Freq	100%	Infreq BC and mow; recent herb

* Doubled at least during the study period due to brush cutting and tree clearing; ** grassland area estimated for entire Thomson complex; *** only includes this site in Buena Vista Grassland site complex; **** core area burned in 1991, then again in 2011. Abbreviations for frequency: Freq (frequent) <5 years return interval; Mod (moderate) 5–10 years return interval; Infreq (infrequent) >10 years return interval; Zero (no burning). Abbreviations for managements: BC, brush cutting; F, fire; CG, cow grazing; CHG, cow grazing with a few horses; GG, goat grazing in rotation of small paddocks; Hay, (mowing with clippings removed); Herb, (herbiciding of brush and/or herbs); Idle, (no management at all); Mow, (mowing with clippings left on ground).

## Appendix A

**Table A1:** Sites of plausible habitat surveyed during 2000–2015 during the expected flight period for that area, but with no regal fritillaries recorded. This is additive to the compilation in [32].

Habitat	Site	County	Years Surveyed
Barrens	Crex Meadows	Burnett	1991–2015
	Dike 17	Jackson	1988–2015
	Sandhill Wildlife Area	Wood	1992–1993, 1995–2015
	Fish Lake Wildlife Area	Burnett	1998–2015
Grasslands	Battle Bluff Prairie	Vernon	2007–2008, 2010, 2013–2014
	Chaffee Creek	Marquette	2000–2002, 2004–2007, 2010, 2012–2015
	Dewey Heights Prairie	Grant	2001–2015
	Governor Dodge State Park	Iowa	2000–2001
	Grand River Wildlife Area	Marquette/Green Lake	2014
	Hardscrabble Prairie	Lafayette	2006–2008
	Leola Wildlife Area	Adams	2000, 2008, 2010, 2012–2015
	Marbleseed Prairie	Green	2001–2008, 2010, 2012–2013
	Puchyan Prairie	Green Lake	2001–2002, 2005–2007, 2009–2015
	Semrad Slough	Grant	2012–2014
	St. Croix County site 1 *	St. Croix	2000
	St. Croix County sites 2–3, 6–8, 11	St. Croix	2000
	White River Wildlife Area	Green Lake	2001–2015
Heaths	Douglas County Wildlife Area	Douglas	2003, 2005, 2011–2015
	Dunbar Barrens	Marinette	2002–2011
	Marinette County Forest	Marinette	2002–2014
	Moquah	Bayfield	2001–2002, 2004–2015
	Spread Eagle	Florence	2002–2003, 2005–2011

* 3 Regal fritillary individuals found in 1998, 1 in 1999.

## Appendix B. Literature Citations of 1977–2015 4th of July (NABA) Butterfly Count Program

- Hathaway, M. (Ed.) *1977 Butterfly Count Report*, *Wings*; The Xerces Society: Portland: OR, USA, 1977; Volume 4, pp. 1–11.
- Hathaway, M. (Ed.) *1977 Butterfly Count Report!!! Wings;* The Xerces Society: Portland: OR, USA 1978; Volume 4 and 5, pp. 4–10.
- Hathaway, M. (Ed.) *1978 Fourth of July Butterfly Count Report*, *Wings*; The Xerces Society: Portland: OR, USA; 1978, Volume 5, pp. 7–11.
- Heller, I. *1980 Butterfly Count Results*; Atala Supplement Volume 8; The Xerces Society: Portland: OR, USA, 1980 [1982].
- Heller, I. *1981 Butterfly Count Results*; Atala Supplement Volume 8; The Xerces Society: Portland: OR, USA, 1980 [1982].
- Opler, P.A.; Brown, J.W. (Eds.) *Butterfly Counts 1987*; Supplement to Atala Volume 16; The Xerces Society: Portland, OR, USA, 1988.
- Opler, P.A.; Brown, J.W. (Eds.) *Fourth of July Butterfly Counts 1988 Report*; The Xerces Society: Portland, OR, USA, 1989.
- Opler, P.A.; Brown, J.W. (Eds.) *Fourth of July Butterfly Counts 1989 Report*; The Xerces Society: Portland, OR, USA, 1990.
- Opler, P.A.; Brown, J.W. (Eds.) *Fourth of July Butterfly Counts 1990 Report*; The Xerces Society: Portland, OR, USA, 1991.
- Opler, P.A.; Powell, J.A. (Eds.) *Butterfly Counts 1982 & 1983*; The Xerces Society: Portland, OR, USA, 1984.
- Opler, P.A.; Powell, J.A. (Eds.) *Butterfly Counts 1984*; The Xerces Society: Portland, OR, USA, 1985.
- Opler, P.A.; Powell, J.A. (Eds.) *Butterfly Counts 1985*; Supplement to Atala Volume 14; The Xerces Society: Portland, OR, USA, 1986.
- Opler, P.A.; Powell, J.A. (Eds.) *Butterfly Counts 1986*; Supplement to Atala Volume 15; The Xerces Society: Portland, OR, USA, 1987.
- Opler, P.A.; Swengel, A.B. (Eds.) *Fourth of July Butterfly Counts 1991 Report*; The Xerces Society: Portland, OR, USA, 1992.
- Opler, P.A.; Swengel, A.B. (Eds.) *NABA-Xerces Fourth of July Butterfly Counts 1993 Report*; North American Butterfly Association: Morristown, NJ, USA, 1994.
- Powell, J.A.; Sorenson, J.T. (Eds.) *1979 Butterfly Count Results*; Supplement to Atala Volume 7 (August 1980); The Xerces Society: Berkeley, CA, USA, 1980.
- Pyle, S. *Report of the Xerces Society 1st Annual Fourth of July Butterfly Count*; Atala Supplement Volume 3; The Xerces Society: Berkeley, CA, USA, 1975; pp. 38–41.
- Swengel, A.B. (Ed.) *2001 Report NABA Butterfly Counts*; North American Butterfly Association: Morristown, NJ, USA, 2002.
- Swengel, A.B.; Opler, P.A. (Eds.) *Fourth of July Butterfly Counts 1992 Report*; The Xerces Society: Portland, OR, USA, 1993.
- Swengel, A.B.; Opler, P.A. (Eds.) *NABA-Xerces Fourth of July Butterfly Counts 1994 Report*; North American Butterfly Association: Morristown, NJ, USA, 1995.
- Swengel, A.B.; Opler, P.A. (Eds.) *NABA-Xerces Fourth of July Butterfly Counts 1995 Report*; North American Butterfly Association: Morristown, NJ, USA, 1996.
- Swengel, A.B.; Opler, P.A. (Eds.) *NABA Fourth of July Butterfly Counts 1996 Report*; North American Butterfly Association: Morristown, NJ, USA, 1997.
- Swengel, A.B.; Opler, P.A. (Eds.) *NABA Fourth of July Butterfly Counts 1997 Report*; North American Butterfly Association: Morristown, NJ, USA, 1998.
- Swengel, A.B.; Opler, P.A. (Eds.) *NABA Fourth of July Butterfly Counts 1998 Report*; North American Butterfly Association: Morristown, NJ, USA, 1999.
- Swengel, A.B.; Opler, P.A. (Eds.) *NABA Fourth of July Butterfly Counts 1999 Report*; North American Butterfly Association: Morristown, NJ, USA, 2000.
- Swengel, A.B.; Opler, P.A. (Eds.) *2000 Report NABA Fourth of July Butterfly Counts*; North American Butterfly Association: Morristown, NJ, USA, 2001.
- Swengel, A.B.; Swengel, S.R. (Eds.) *2002 Report NABA Butterfly Counts*; North American Butterfly Association: Morristown, NJ, USA, 2003.
- Swengel, A.B.; Swengel, S.R. (Eds.) *2003 Report NABA Butterfly Counts*; North American Butterfly Association: Morristown, NJ, USA, 2004.
- Swengel, A.B.; Swengel, S.R. (Eds.) *2004 Report NABA Butterfly Counts*; North American Butterfly Association: Morristown, NJ, USA, 2005.
- Wander, S. (Ed.) *2005 Report NABA Butterfly Counts*; North American Butterfly Association: Morristown, NJ, USA, 2006.
- Wander, S. (Ed.) *2006 Report NABA Butterfly Counts*; North American Butterfly Association: Morristown, NJ, USA, 2007.
- Wander, S. (Ed.) *2007 Report NABA Butterfly Counts*; North American Butterfly Association: Morristown, NJ, USA, 2008.
- Wander, S. (Ed.) *2008 Report NABA Butterfly Counts*; North American Butterfly Association: Morristown, NJ, USA, 2009.
- Wander, S. (Ed.) *2009 Report NABA Butterfly Counts*; North American Butterfly Association: Morristown, NJ, USA, 2010.
- Wander, S. (Ed.) *2010 Report NABA Butterfly Counts*; North American Butterfly Association: Morristown, NJ, USA, 2011.
- Wander, S. (Ed.) *2011 Report NABA Butterfly Counts*; North American Butterfly Association: Morristown, NJ, USA, 2012.
- Wander, S. (Ed.) *2012 Report NABA Butterfly Counts*; North American Butterfly Association: Morristown, NJ, USA, 2013.
- Wander, S. (Ed.) *2013 Report NABA Butterfly Counts*; North American Butterfly Association: Morristown, NJ, USA, 2014.
- Wander, S. (Ed.) *2014 Report NABA Butterfly Counts*; North American Butterfly Association: Morristown, NJ, USA, 2015.
- Wander, S. (Ed.) *2015 Report NABA Butterfly Counts*; North American Butterfly Association: Morristown, NJ, USA, 2016.
